# Estimation of the amount of pear pollen based on flowering stage detection using deep learning

**DOI:** 10.1038/s41598-024-63611-w

**Published:** 2024-06-07

**Authors:** Keita Endo, Takefumi Hiraguri, Tomotaka Kimura, Hiroyuki Shimizu, Tomohito Shimada, Akane Shibasaki, Chisa Suzuki, Ryota Fujinuma, Yoshihiro Takemura

**Affiliations:** 1 Nippon Institute of Technology, Saitama, 345-8501 Japan; 2 Faculty of Science and Engineering, Doshisha University, Kyoto, 610-0321 Japan; 3 Saitama Agricultural Technology Research Center, Saitama, 346-0037 Japan; 4 Saitama Agriculture and Forestry Promotion Center, Saitama, 330-0074 Japan; 5 DKK Co., Ltd., Tokyo, 100-0005 Japan; 6 Faculty of Agriculture, Tottori University, Tottori, 680-8550 Japan

**Keywords:** Computer science, Image processing, Machine learning, Agroecology

## Abstract

Pear pollination is performed by artificial pollination because the pollination rate through insect pollination is not stable. Pollen must be collected to secure sufficient pollen for artificial pollination. However, recently, collecting sufficient amounts of pollen in Japan has become difficult, resulting in increased imports from overseas. To solve this problem, improving the efficiency of pollen collection and strengthening the domestic supply and demand system is necessary. In this study, we proposed an Artificial Intelligence (AI)-based method to estimate the amount of pear pollen. The proposed method used a deep learning-based object detection algorithm, You Only Look Once (YOLO), to classify and detect flower shapes in five stages, from bud to flowering, and to estimate the pollen amount. In this study, the performance of the proposed method was discussed by analyzing the accuracy and error of classification for multiple flower varieties. Although this study only discussed the performance of estimating the amount of pollen collected, in the future, we aim to establish a technique for estimating the time of maximum pollen collection using the method proposed in this study.

## Introduction

Among fruit trees, pears, except for some pear varieties, are characterized by self-incompatibility and require pollen from other varieties to bear fruit. For pollination, planting several adjacent pear varieties is desirable to create a pollinating environment in which insects (honeybees) can be used as vectors. However, the flowering period of pears is from late March to early April, and insect activity often decreases when temperatures are low and rainfall continues during this period, resulting in unstable pollination and fruit set. Therefore, growers use artificial pollination as a reliable method of stabilizing fruit sets^1–3^. Artificial pollination requires pollen collection and preparation by growers. Pollen collection requires long hours of manual labor because pollen is collected from flowering pear branches at high elevations using stepladders. As a result, the percentage of imported pollen has recently increased. However, the high cost of purchasing imported pollen and the inability to secure sufficient amounts can significantly reduce production^4,5^. To address these problems, improving the efficiency of pollen collection and strengthening the systems and mechanisms of domestic supply and demand is essential.

Smart agricultural methods incorporating Artificial Intelligence (AI) and other information technologies are being actively promoted in Japan by the government, academia, and the private sector to reduce the labor required to support farming operations, grow high-quality crops, and increase production^6^. Smart agriculture is also beginning to be defined as a new technology that extends information and communication field technologies such as the Internet of Agro-Things (IoAT) and Agro-Cyber Physical Systems (A-CPS) to agriculture^7–9^. For example, AI-based optimal irrigation control technology has been proposed and tested for the cultivation of tomatoes with high sugar content^10–12^. Another example of a practical application is hydroponic cultivation used in plant factories, which is useful for cultivation conditions that can be largely artificially controlled, such as optimizing light intensity using LED artificial lighting^13–15^. In addition, studies are being conducted on robots that assist in the cultivation of crops in place of humans to save labor. Applications of robot-assisted cultivation technology, such as the automation of pesticide applications for pest control^16–18^ and crop harvesting^19–21^. However, robots will be required to make decisions and perform analyses instead of human operators. Recently, AI-based image analysis has attracted considerable attention, and various image analysis technologies have been used^22–24^.

In this study, we proposed an AI pear pollen amount estimation technology using deep learning, a type of AI, to maximize the collection of pear pollen and mechanize the pollen collection process. As part of the technique for estimating when the maximum amount of pollen can be collected, this study described a technique for calculating the amount of pollen that can be collected using deep learning to determine the shape of a pear flower on an image of a blooming branch. We used You Only Look Once (YOLO)^25–27^ for classifying and detecting the shape of each flower using deep learning. YOLO is an object-detection algorithm based on deep learning that uses neural networks to analyze images. First, an image of a branch with flowers was captured using a camera, and the captured image was resized to simplify analysis using deep learning. Next, small square grid cells were created in the resized image and trained to classify objects using the grid cells. As a previous study, there is a paper^28^ that estimates the peak flowering period of pear blossoms based on temperature changes. However, there are no studies that estimate pollen amounts or the optimal pollen collection time using AI. In the aforementioned paper^28^ estimating the peak flowering period of pear blossoms based on temperature changes, accumulation is done based on temperature changes, making real-time corrections impossible. On the other hand, with the method of this study, estimation can be conducted in real-time by capturing images every day while making adjustments. In this study, we report on the development of an AI pear pollen amount estimation technology that estimates the amount of pollen that can be collected based on the number of flowers per flower shape classified by deep learning and the amount of pollen collected per flower calculated by actually collecting pollen from each flower shape. We also report on the evaluation of the estimation accuracy of the developed AI pear pollen amount estimation technology.

## Methodology

YOLO was used to identify pear flower shapes and estimate the amount of pollen. Pollen samples were collected from different flower shapes, and the amount of pollen collected per flower for each flower shape was calculated. The number of flowers of each flower shape detected by YOLO was multiplied by the amount of pollen collected per flower for each flower shape and added to estimate the total amount of pollen.

### Pollen collection according to flower shape

**Figure 1:**
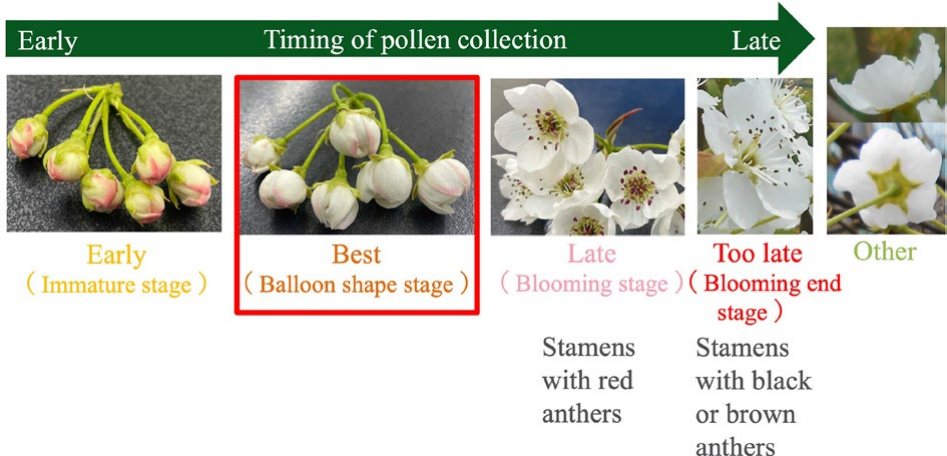
Flowering stages of pear flowers.

Flowers were classified into five shapes, from bud to post-bloom as flowering stages, as shown in Fig. 1. The flowering stages were:“immature stage”, “balloon shape stage”, “blooming stage”, and “blooming end stage”, in which the pollen had fallen off after blooming, and “other”, in which the sides and back of the flower were shown and the “blooming stage” and “blooming end stage” were indistinguishable. “Blooming stage” and “blooming end stage” both have flowers in full bloom, but the anthers of the stamens were red in the “blooming stage”, whereas the anthers of the stamens turned black or brown in the“blooming end stage”.

In Table 1, flowers of three pear varieties, Chojuro, Shinko, and Nepal, were collected for each flowering stage in 100 flower $\times$ 3 replications, and the pollen amount collected per flower was calculated from the total pollen amount and the number of flowers in each flowering stage. As an example, the standard deviation of Chojuro was $\mathrm {9.428 \times 10^{-6}}$ for Early, $\mathrm {1.247 \times 10^{-5}}$ for Best, and $\mathrm {1.247 \times 10^{-5}}$ for Late for each flowering stage, indicating that the variation was small enough to calculate the pollen amount per flower with 100 flowers $\times$ 3 replications. To collect and weigh pollen from flowers, collected flowers were placed in anther-extracting machine, petals were removed, and anthers were sieved to remove only stamens. A high precision anther-extracting machine was then used to remove impurities. Anthers collected by these procedures were placed in an anther opening machine and weighed after anther opening. The amount of pollen collected per flower was 0 mg at the “blooming end stage” because pollen was almost impossible to collect due to pollen bursting. Specifically, the “blooming end stage” was excluded from the estimated pollen amount, although it was included in the five types of classification even if pollen could not be collected. The amount of pollen collected per flower in “other” was calculated variably based on the ratio of the “blooming stage” to the “blooming end stage” in the image taken. The effective pollen amount in Table 1 is the pollen amount that takes into account the viability of pollen per flowering stage by calculating the fruiting rate, which is the probability of successful pollination by artificial pollination using pollen from each collected flowering stage, and multiplying the pollen amount per flower by the fruiting rate. The amount of pollen collected per flower at each stage was the highest in the balloon-shaped flowering stage, although it varied depending on the species. Based on these results, the “immature stage” was called “Early”, the “balloon shape stage” was “Best”, the “blooming stage” was “Late”, the “blooming end stage” was “Too late”, and flowers that could not be distinguished between the “blooming stage” and the “blooming end stage” were “Other”. The AI pollen amount estimation by AI was performed using pollen amount data per flower, as shown in Table 1. However, because the proportion of flowering stages and the number of flowers differed for each flower cluster or branch, we developed an AI that could identify the number of flowers at each flowering stage by training image data obtained randomly from branches during the flowering stage using YOLO.

**Table 1:** Amount of pollen collected per flower at each flowering stage of each variety.

Flowering stage	Amount of pollen collected per flower (mg)			Effective pollen amount (mg)		
	Chojuro	Shinko	Nepal	Chojuro	Shinko	Nepal
Early	0.367	0.960	0.902	0.201	0.671	0.428
Best	0.757	1.347	1.137	0.386	0.871	0.795
Late	0.387	0.720	1.141	0.257	0.545	0.783
Too late	0	0	0	0	0	0

### Flowering stage classification method using YOLO

YOLO, an object-detection algorithm based on deep learning, was used as the AI method. The number of images of flowers captured at random was 1271, and the flowering stage was learned by annotating the images. For annotation, flower parts were extracted from the image data of flower clusters and branches, and five types of labels (“Early”, “Best”, “Late” ,“Too late”, and “Other”) were assigned to each flowering stage. The annotated image data were used for training using YOLO.

**Figure 2:**
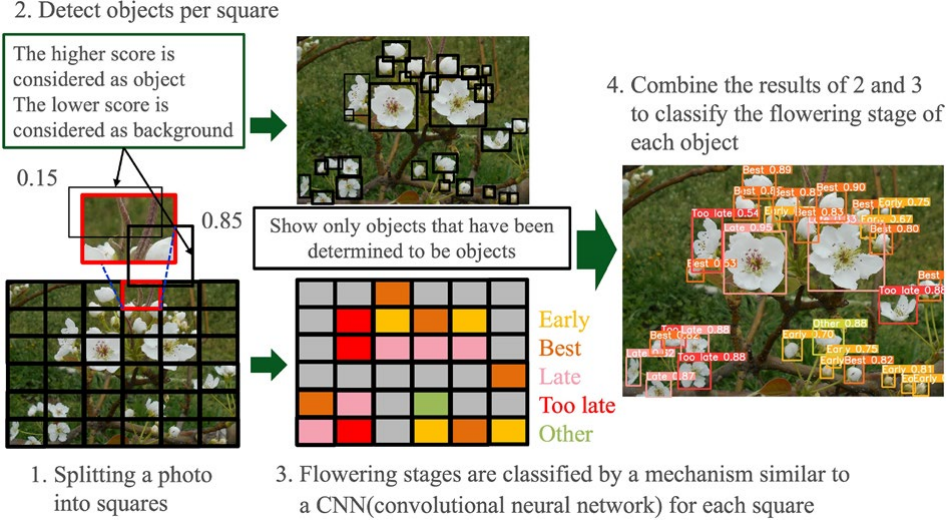
Flower detection and flowering stage identification algorithm using YOLO.

The flower detection and flowering stage identification algorithm using YOLO are illustrated in Fig. 2. To perform object detection, YOLO identified the flowers that appeared in an image. The detection procedure consisted of inputting an image captured by a camera, detecting objects using AI image analysis, and outputting the detection results. Specifically, the results of where the “Early” object in the image was reflected were output almost in real time. First, the input image was divided into small squares (grid cells). Each of these squares was further populated with rectangular bounding boxes. The area enclosed by the bounding boxes was then identified as a background or object. The degree of discrimination between the background and the object was quantified in the range $0 \leqq confidence \leqq 1$. In contrast, each segment divided by a small square was determined by the class to which it belonged. In the example shown in the figure,“Early” is yellow, “Best” is orange, “Late” is pink, “Too late” is red, “Other” is yellowish green, and the rest of the background is gray, for a total of six to classify. Based on these results, a Non-Maximum Suppression (NMS) method was used to extract bounding boxes that were prominently classified into different classes (labels). First, NMS selected the box with the highest confidence level for each class. Subsequently, the Intersection over Union (IoU) of the overlap between the bounding box and other bounding boxes was checked, and bounding boxes that overlapped by more than a certain percentage were eliminated to complete flower detection and shape identification using YOLO.

In this study, 1017 of the 1271 images were used for training and 254 for validation and testing. The image size was 640 $\times$ 640 pixels. The pear varieties in the images were mainly Chojuro, Hosui, Shinko, and Matsushima and were used for training. The batch size for training was set to 16, and 400 epochs to obtain sufficient discrimination accuracy. The YOLOv8n model was used as the training model base, and Adam was used as the optimizer. The number of flowers at each flowering stage was determined by inputting test image data into the model trained under these conditions. The amount of pollen per branch or flower cluster was calculated based on these results. Based on the amount of pollen collected per flower obtained in Table 1 and the YOLO detection results, we calculated “the number of flowers detected by YOLO” $\times$ “the amount of pollen collected per flower” for each flowering stage and added them together to obtain the estimated pollen amount. To estimate the overall pollen amount from the entire tree, one can take multiple photos and derive the estimated pollen amount from the sum of the detected results.

### Ethics declarations

The use of plants in this study complied with relevant institutional, national, and international guidelines and legislation.

## Results

### Evaluation of the YOLOv8 training model

**Figure 3:**
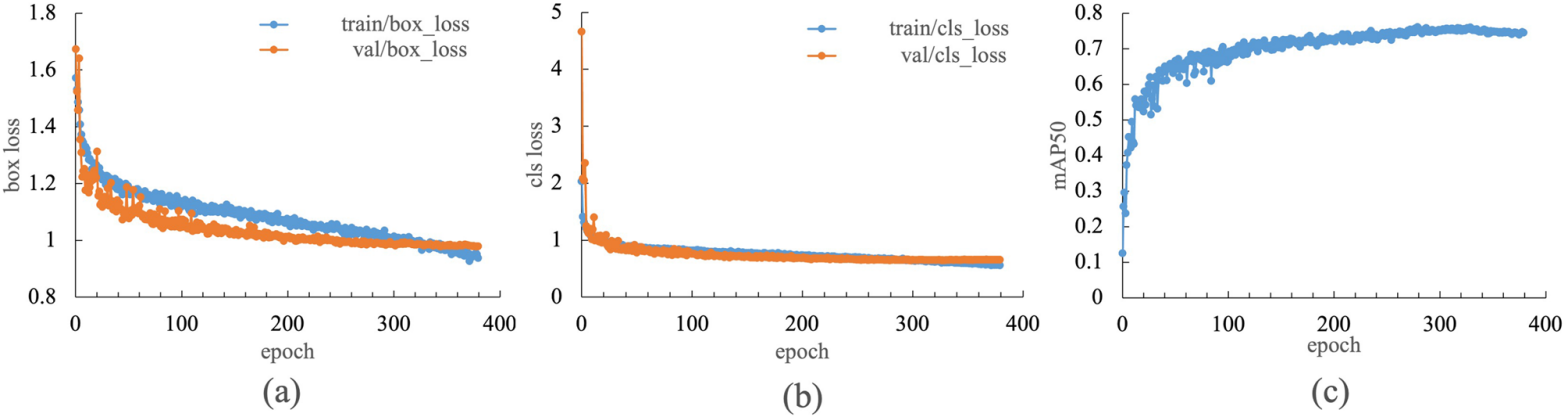
Various evaluations of the training model of YOLOv8. (a) Loss rate for detection frame. (b) Loss rate for detection class. (c) mAP50.

Figure 3 shows various evaluations of the YOLOv8 model trained on the flowering stages of pears. Fig. 3a is a graph of the loss rate for detection boxes (box loss). For example, the detection box is illustrated in Fig. 2 when each flower was detected. Fig. 3b is a graph of the loss rate for detection classes (class loss). These two graphs show that as the training progresses, the slope of val, which is the evaluation when the validation image is detected, becomes gentler, indicating that the training is converging. In addition, the values of train, which was the evaluation when the training image was detected, and val, which was the evaluation when the validation image was detected, were close. Therefore, training was completed normally without overfitting. At 380 epochs, Early Stopping was performed to prevent overfitting, and the training was completed. Figure 3c shows the mAP50 (mean Average Precision)^29–31^ graph. mAP is an index used to evaluate the accuracy of object detection, such as YOLO. The mAP50 was 0.76 at the 330th epoch; therefore, the training model at the 330th epoch was used to estimate the amount of pollen.

### Example of detection results and detection accuracy for each flowering stage using YOLOv8

**Figure 4:**
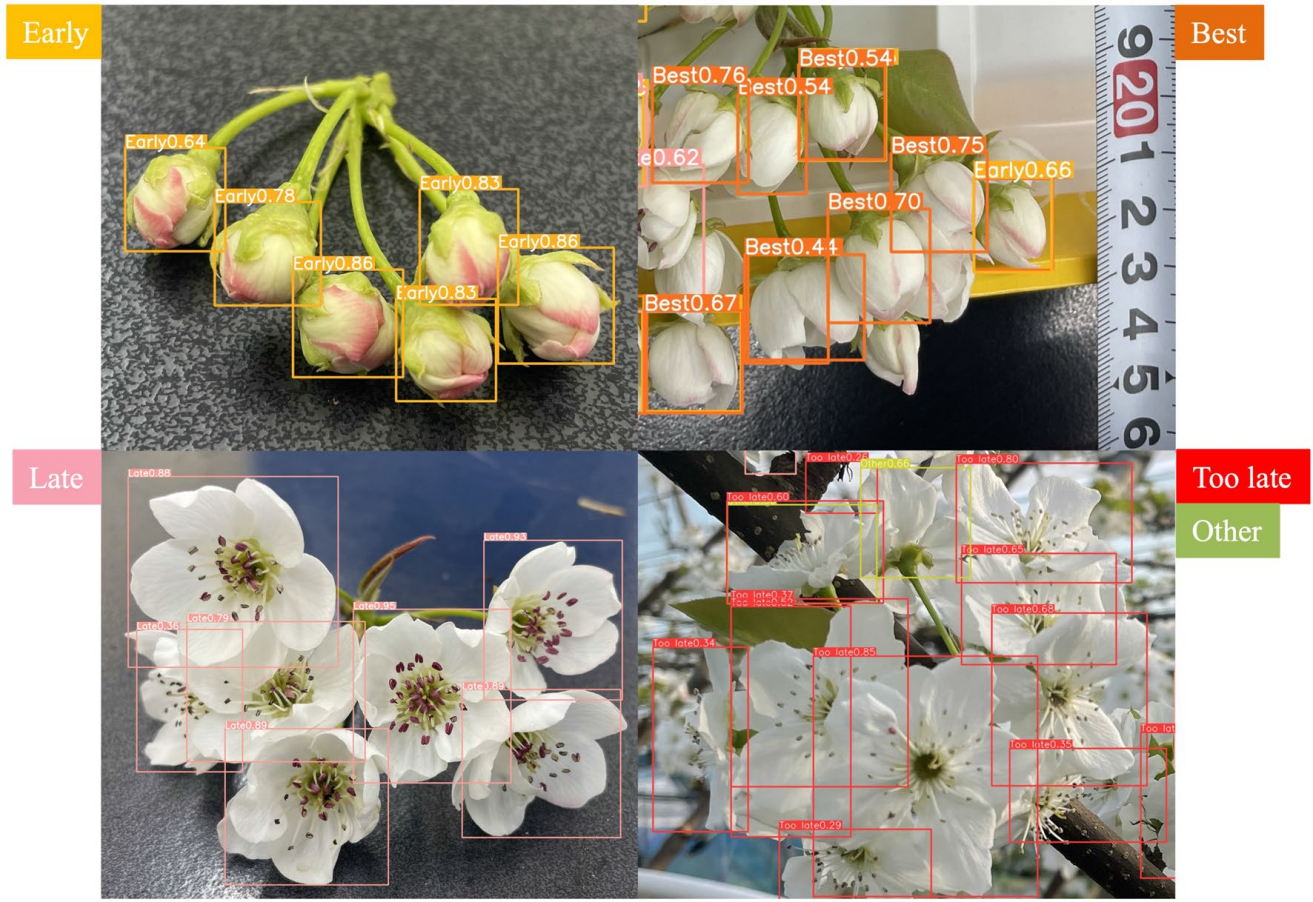
Example of detection results of each flowering stage using YOLOv8.

**Figure 5:**
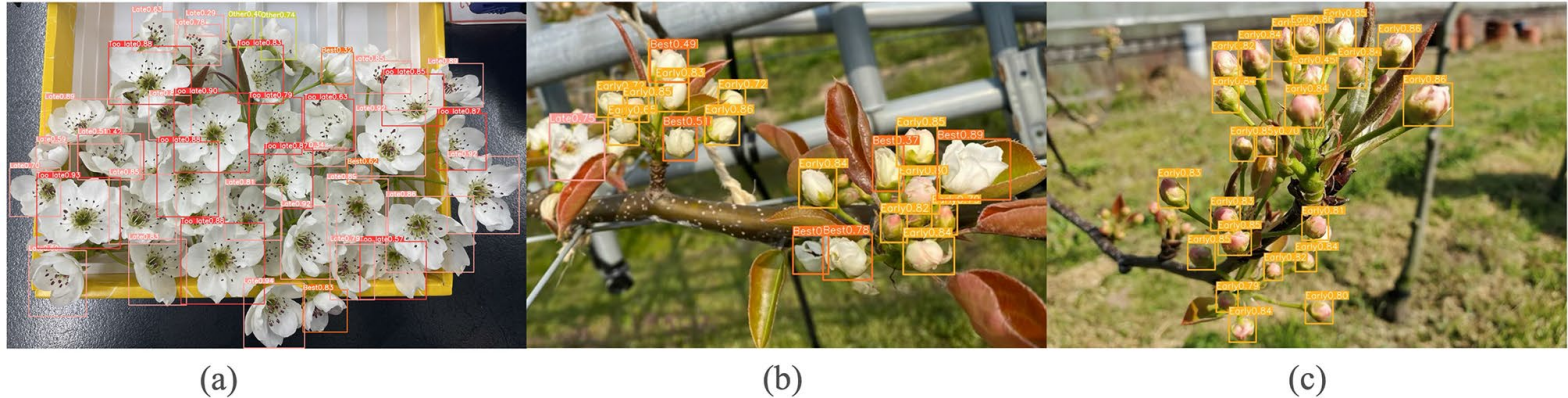
YOLOv8 detection results for images with mixed flowering stages. (a) Late, Too late and Other mixed. (b) Early and Best mixed. (c) Early.

**Figure 6:**
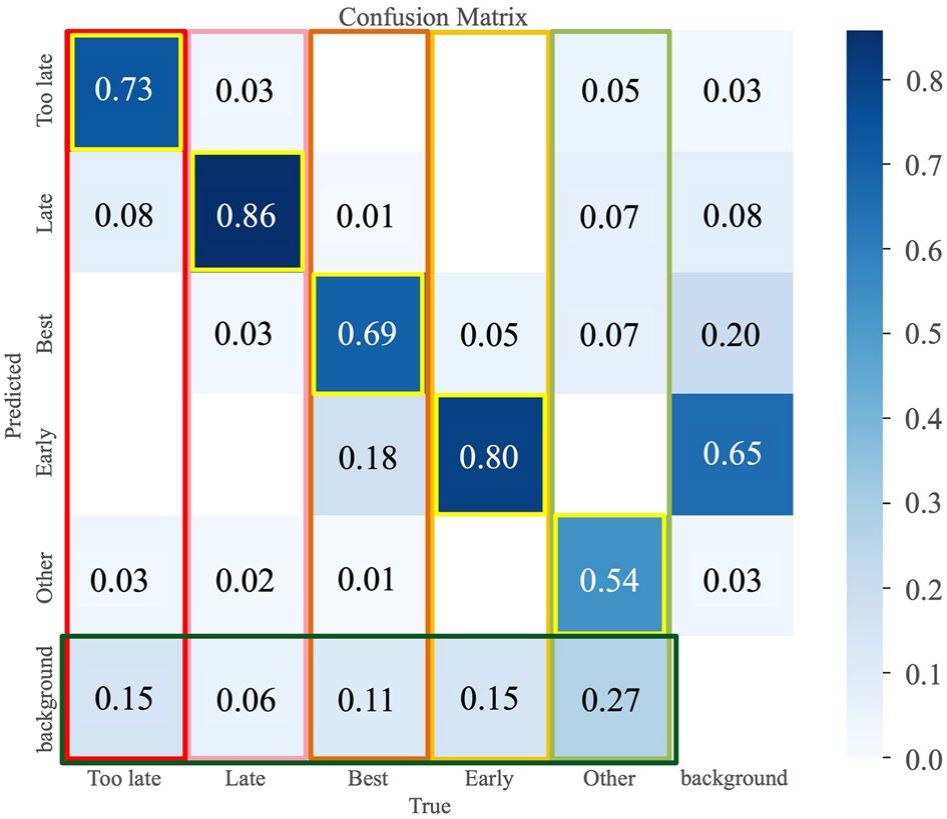
Detection accuracy for each flowering stage.

Figure 4 shows an example of the detection results of each flowering stage using YOLOv8. “Early” was detected with a yellow frame, “Best” with an orange frame, “Late” with a pink frame, “Too late” with a red frame, and “Other” with a yellow-green frame. The images of the detection results shown in Fig. 4 show that the labels “Early”, “Best”, “Late”, “Too late”, and “Other” were displayed and accurately classified. Figure 5 shows the detection results when flowering stages were mixed. Table 2 lists the results of the accuracy rate calculations for the image shown in Fig. 5, which includes a mixture of “Late”, “Too late”, and “Other”, a mixture of “Early” and “Best”, and an only “Early”. The accuracy rate was calculated as the ratio of the number of correct flowering stage identifications to the number of flowers detected in the image. The flowering stage classifier developed by YOLO can classify flowers with mixed flowering stages with an accuracy of over 80%. However, under the mixed condition of “Late”, “Too late”, and “Other”, the shape of “Late” and “Too late” were almost the same, and the only difference in classification was the color of the anthers, which may have affected the accuracy of classification. However, if the shapes were evidently different, classification could be performed with high accuracy. Figure 6 shows the detection accuracy of YOLOv8 when classifying the flowering stages for 254 images, which were selected for testing and validation of the training model and classification accuracy out of the 1271 images used to create the training model. The validation and test images include images of multiple fields and varieties, as well as evaluations of different environmental conditions. Figure 6 evaluates the classification accuracy, such as the accuracy rate for each flowering stage, the percentage of misclassified flowering stages, and the detection accuracy, such as the degree to which flowers in each flowering stage are detected. The vertical columns in Fig. 6 show the correct (True) flowering stages, and the horizontal rows show the flowering stages estimated by YOLOv8 (Predicted). The flowers at each flowering stage are shown in the vertical columns; the flowering stage classified by YOLOv8 is shown in the horizontal rows, and the proportion is shown at the intersection of the vertical and horizontal columns. Therefore, the yellow box, where the True and Predicted flowering stages coincide, indicates the accuracy rate for each flowering stage. The accuracy rate for each flowering stage, “Early” and “Late”, were 80% and 86%, respectively, which was a high accuracy rate. In contrast, the accuracy rates for “Best” and “Other” were low at 69% and 54%. For the “Best” column, there was an 18% rate of misclassifying “Best” as “Early”. This suggests that the similarity of the flower shape between “Best” and “Early” may have caused the misclassification, resulting in a lower accuracy rate for “Best”. The background frame indicates the percentage of undetectable flowers not included in the evaluation in Table 2. From the ratio of “Early”, “Too late”, and “Other” backgrounds, the accuracy rate decreased due to a higher proportion of flowers not being detected than to misclassification of the flowering stage. In particular, the decrease in the accuracy rate for “Other” is considered to be due to the aforementioned large percentage of undetected flowers. To solve these problems, the detection accuracy can be improved by increasing the number of training images by extracting image data that are difficult to misclassify or detect. A solution to the problem of improving detection accuracy will be addressed in future work.

**Table 2:** Accuracy rate for the images in Fig. 5.

Accuracy rate (%)		
Late, Too late and Other mixed	Early and Best mixed	Early
82.05	89.47	100

### Example of pollen amount estimation results

**Figure 7:**
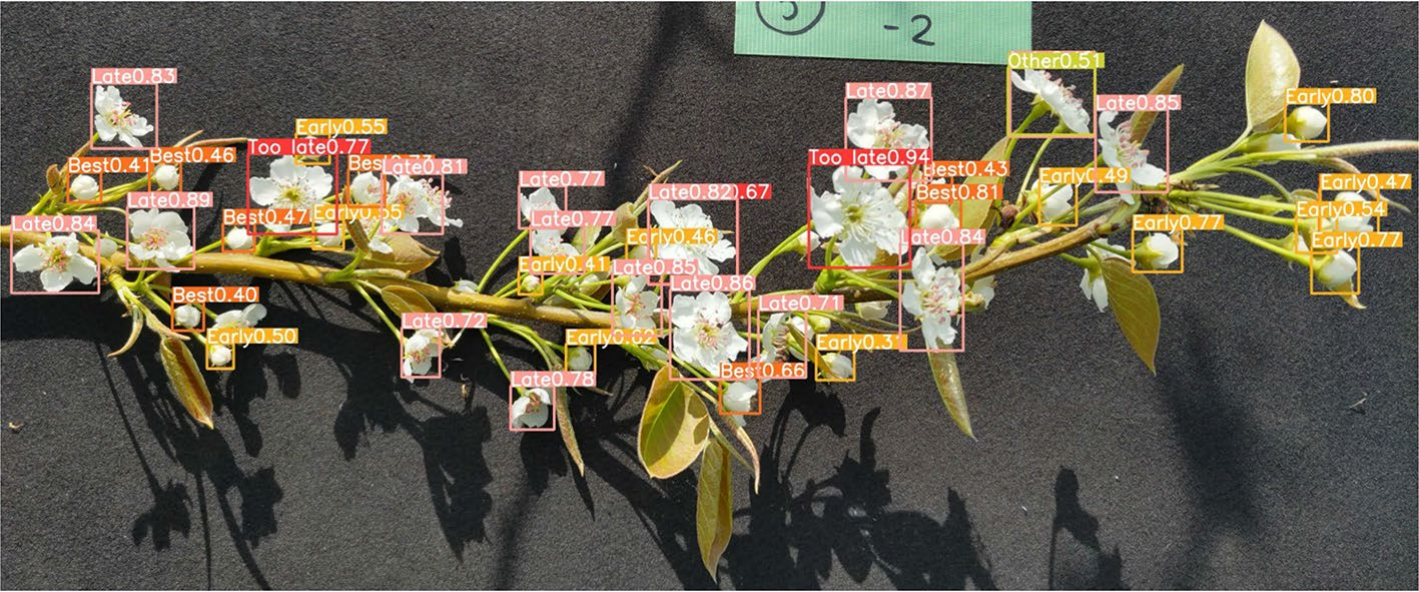
Image of flowering stages detected by YOLOv8.

Figure 7 shows an image of flowering stages detected by YOLOv8 using the 330th epoch model described above. For example, the flowering stage was detected in an image of a branch of the Chojuro pear variety. “Early” was detected in yellow, “Best” in orange, “Late” in pink, “Too late” in red, and “Other” in yellow-green. The flowering stage was generally correctly detected from the images of the detection result. In addition, the reliability of the output next to the label was generally high, and the flowering stage could be detected.

Table 3 lists the results of the pollen amount estimation for the detection results shown in Fig. 7. The estimated pollen amount was obtained by calculating “the number of flowers detected by YOLOv8” $\times$ “the amount of pollen collected per flower” for each flowering stage and adding up the results of the calculations for each stage. The detection results are presented in Fig. 7, and the estimated pollen amount was 18.102 mg. In contrast, the measured amount of pollen collected from flowers on the branch was 55.0 mg. Although it is desirable for the estimated pollen amount to be the same as the actual pollen amount, the comparison showed an error. The causes of these errors are discussed in the following sections.

**Table 3:** Results of estimating pollen amount for Fig. 7.

Flowering stage	Number of flowers detected by YOLOv8	Amount of pollen collected per flower (mg)	Estimated pollen amount by YOLOv8 (mg)
Early	13	0.367	4.771
Best	9	0.757	6.813
Late	16	0.387	6.192
Too late	3	0.000	0
Other	1	Late and Too late ratio to be determined	0.326
Sum total	42		18.102

## Discussion

The causes of the errors between the estimated and actual pollen amounts can be summarized into the following three categories:

#1: Error due to the amount of pollen collected per flower.
  The error is because the average amount of pollen collected per flower was not collected from a sufficient number of pear flowers and also due to the misclassification of flowers into each flowering stage by the human eye
#2: Error due to inability to detect flowers hidden behind branches.
  ﻿Cameras can only take flat images; therefore, if a flower is hidden behind a branch or stem, it will not appear in the image, and YOLOv8 cannot detect it, causing errors.
#3: Error due to YOLOv8’s decision accuracy.
  These errors are caused by flowers that could not be detected by YOLOv8 and the misclassification of flowering stages by YOLOv8.

### Accuracy of pollen amount estimation

To evaluate the accuracy of pollen estimation, we discuss the causes of the error between the estimated and actual pollen amounts described above.

Considering #1 in the previous section. For the branches depicted in Fig. 7, “the actual number of flowers” $\times$ “the amount of pollen collected per flower” was calculated to be 27.65 mg. This value should be the same as the actual pollen amount of 55.0 mg but with an error. Therefore, an error occurred in the amount of pollen collected per flower. Because the amount of pollen collected per flower could not be corrected in this experiment, the error in the estimated pollen amount was calculated using Formula (1), which considers the error in the amount of pollen collected per flower.

1 $$\begin{aligned} \text {Error in estimated pollen amount} (\%)&=-\left( 1-\frac{\text {Estimated pollen amount by YOLOv8}}{\text {Actual number of flowers times Pollen collected per flower}}\right) \times 100 \nonumber \\&=-\left( 1-\frac{\text {Number of flowers detected by YOLOv8}}{\text {Actual number of flowers}}\right) \times 100 \end{aligned}$$

Pollen amounts were estimated by preparing at least 10 images for each of the three varieties (Chojuro, Shinko, and Nepal), with one flowering branch as the subject, and the actual number of flowers was recorded for each. The error in the estimated pollen amount was calculated by substituting the number of flowers detected by YOLOv8 and the actual number of flowers visually observed, as described above, into Formula (1).

**Figure 8:**
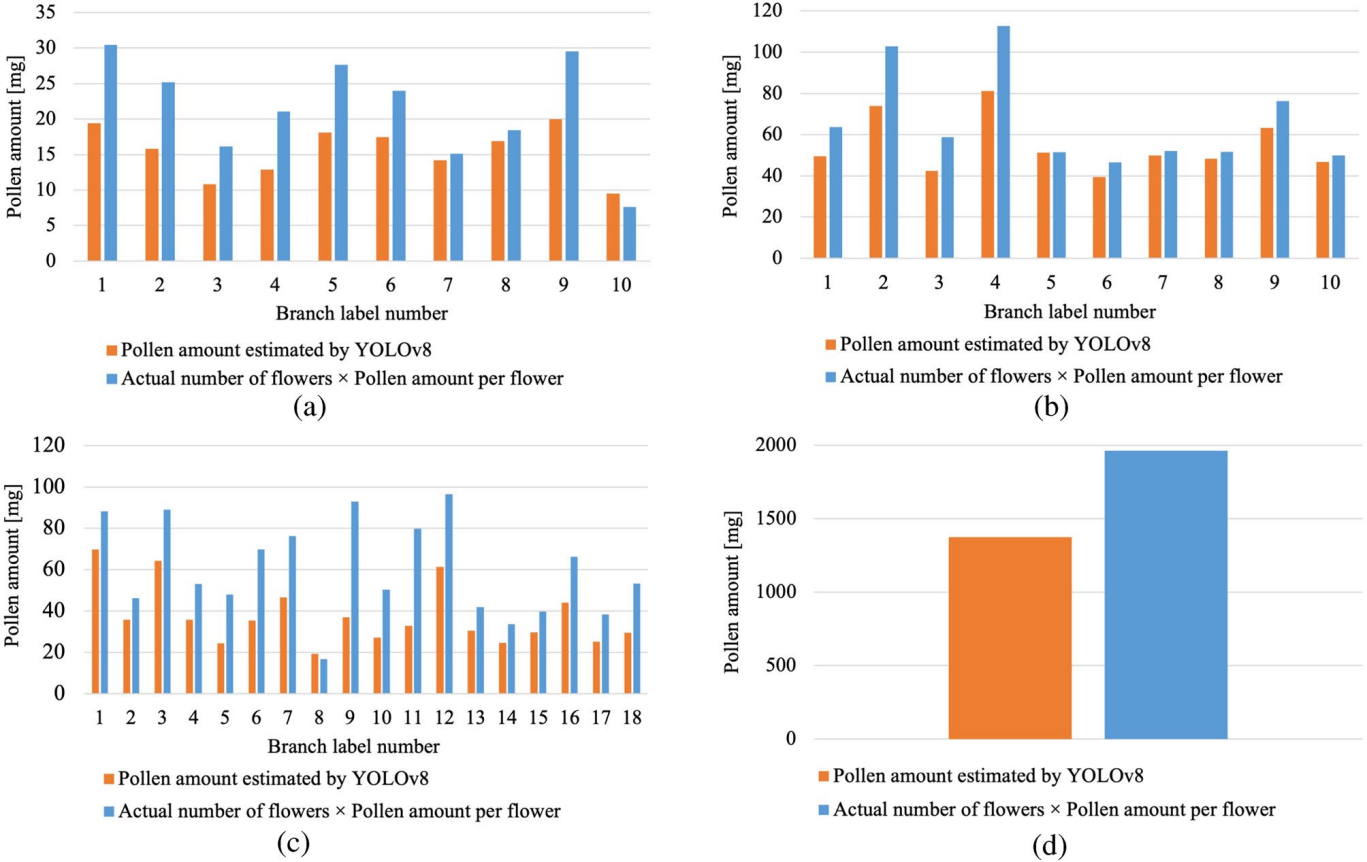
Comparison of estimated pollen amount and actual number of flowers $\times$ amount of pollen collected per flower. (a) Chojuro. (b) Shinko. (c) Nepal. (d) Total of 3 varieties.

**Figure 9:**
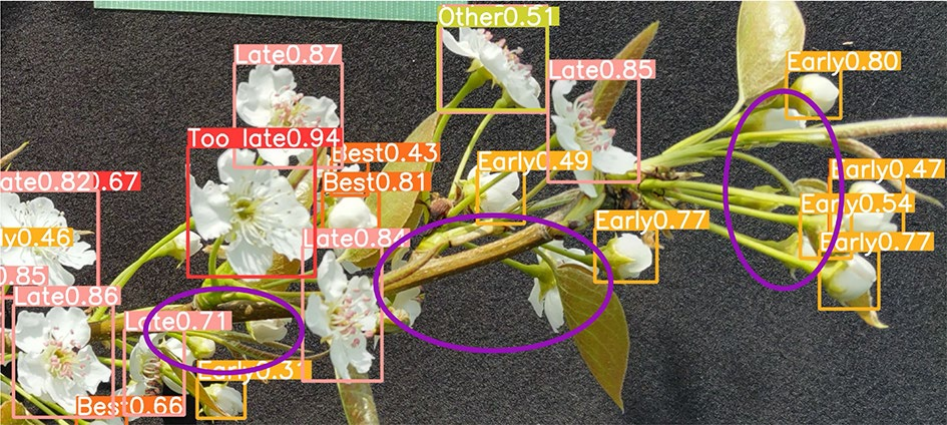
Examples of flowers hidden behind branches and stems.

The errors in the estimated pollen amounts are listed in Table 4, and #2 in the previous section is discussed. Table 4 shows that the errors in the pollen amount estimates varied from − 18.02% to − 37.70%, depending on the variety. The average error for the three varieties was − 27.89%. The error in the estimated pollen amount was negative, indicating that the pollen amount estimated by YOLOv8 was less than “the actual number of flowers” $\times$ “the amount of pollen collected per flower”. Figure 8 shows a graph comparing the estimated pollen amount and “the actual number of flowers” $\times$ “the amount of pollen collected per flower” for each branch of each variety. Figure 8 shows that the error in the estimated pollen amount varied from branch to branch. Therefore, the error in the estimated pollen amount was considered to be caused by the fact that flowers hidden behind the branches could not be detected because they were not captured in the image. Figure 9 shows an example of a flower hidden behind a branch or stem. The purple circle in Fig. 9 shows that the branch or stem obstructs the flower and cannot be detected. Therefore, considering the camera’s viewing angle and assuming that the flowers are hidden at approximately 45°–90°, an error of approximately 12.5–25% is considered reasonable. Therefore, the error could be eliminated by multiplying the estimated pollen amount by 1.125–1.25, which is a parameter for flowers hidden behind the flowers. In this case, the error can be reduced to − 2.89 to − 15.39% by considering the flower parameters hidden behind the pollen.

We considered #3 in the previous section as the cause of the remaining error. The judgment accuracy of YOLOv8 was determined by calculating the accuracy rate using Formula (2).

2 $$\begin{aligned} \text {Accuracy rate} (\%)=\frac{\text {Number of flowers judged correctly by YOLOv8}}{\text {Number of flowers detected by YOLOv8}}\times 100 \end{aligned}$$

YOLOv8 was used to detect the flowering stage in 35-pear flower images. Table 5 lists the accuracy rate for determining the correct flowering stage using (2). Table 5 shows that the accuracy rate varied from 81.481 to 87.903% depending on the variety. The average result for the five varieties was 83.407%, indicating that the flowering stage was detected with high accuracy. However, as the percentage was not 100%, it was considered that some errors occurred in the estimated pollen amounts owing to the accuracy of YOLOv8’s judgment. Table 6 shows the results of the comparison between the percentage of the actual number of flowers per flowering stage and the percentage of the number of flowers estimated by YOLOv8. The reason why “Other” was not included in the measured data was that the actual flower counts were always classified as either “Late” or “Too late” when sorted by the human eye. Table 6 shows that some flowers could not be detected because the number of flowers detected by YOLOv8 was lower than the actual number. However, by comparing the percentage of each flowering stage in the measured data with the percentage of each flowering stage in the YOLOv8 estimate, the error was small, ranging from − 7.1 to 3.9%. In particular, the error for the “Best” flowering stage was 0.4%, indicating that the detection rate for the flowering stage with the most pollen available was correct. Therefore, although the present estimation cannot accurately estimate the pollen amount, it is possible to estimate the day of maximum pollen amount by following the daily transition of pollen amount because the detection rate of each flowering stage was close to the actual measurement value. Therefore, the AI pear pollen amount estimation technology developed in this study can estimate the best time to collect the maximum amount of pollen from pear branches before collection, which is the ultimate goal of the study.

**Table 4:** Error in pollen amount estimated by YOLOv8.

All varieties	Chojuro	Shinko	Nepal
− 27.89 (%)	− 27.94 (%)	− 18.02 (%)	− 37.70 (%)

**Table 5:** Average accuracy rate for YOLOv8.

All varieties	Chojuro	Shinko	Nepal	Matsushima	Yokoyama
83.407 (%)	83.396 (%)	87.903 (%)	84.314 (%)	81.494 (%)	81.481 (%)

**Table 6:** Comparison of flowering stage percentage between actual measured value and estimated value by YOLOv8.

Flowering stage	Actual measured values collected			Estimated values by YOLOv8			Error rate (%)
	Number of flowers	Pollen amount (mg)	Percentage of flowering stages (%)	Number of flowers	Pollen amount (mg)	Percentage of flowering stages (%)	
Early	19	6.973	30.6	13	4.771	31.0	0.4
Best	13	9.841	21.0	9	6.813	21.4	0.4
Late	28	10.836	45.2	16	6.192	38.1	−7.1
Too late	2	0	3.2	3	0	7.1	3.9
Other				1	0.326	2.4	
Sum total	62	27.650		42	18.102		

## Conclusion

In this study, we developed an AI pear pollen amount estimation technology using YOLO to estimate the amount of pollen that can be collected from pear flowers and evaluated its estimation accuracy. We considered three reasons for the error between the pollen amount estimated by YOLOv8 and the actual pollen amount measured: an error in the actual amount of pollen collected per flower, an error owing to the inability to detect flowers hidden behind branches, and an error owing to the accuracy of YOLOv8’s judgment. In the process of evaluating the estimation accuracy, we found that YOLOv8 could estimate the general amount of pollen by considering the error in the actual amount of pollen collected per flower and by parameterizing the flowers hidden behind the branches. Because the percentage of each flowering stage estimated by YOLOv8 was close to the percentage of each flowering stage measured, the accuracy of the AI pear pollen amount estimation technology was high enough to establish the elemental technology for estimating the best time to collect the maximum pollen amount.

In the future, we aim to establish an algorithm that predicts the optimal timing for pollen collection using AI pear pollen amount estimation in combination with trends in temperature change and flowering stages.

## Data Availability

The datasets used and/or analysed during the current study available from the corresponding author on reasonable request.
